# Emerging geo-data sources to reveal human mobility dynamics during COVID-19 pandemic: opportunities and challenges

**DOI:** 10.1007/s43762-021-00022-x

**Published:** 2021-09-26

**Authors:** Xiao Li, Haowen Xu, Xiao Huang, Chenxiao (Atlas) Guo, Yuhao Kang, Xinyue Ye

**Affiliations:** 1 Texas A&M Transportation Institute, 1111 RELLIS Pkwy, Bryan, TX 77807 USA; 2 Oak Ridge National Laboratory, Oak Ridge, TN 37830 USA; 3 Department of Geosciences, University of Arkansas, Fayetteville, AR 72701 USA; 4 Department of Geography, University of Wisconsin-Madison, Madison, WI 53706 USA; 5 Department of Landscape Architecture and Urban Planning, Texas A&M University, College Station, TX 77840 USA; 6 Department of Geography, Texas A&M University, College Station, TX 77840 USA

**Keywords:** Mobility data, Mobile device data, Social media, Connected vehicle, COVID-19

## Abstract

Effectively monitoring the dynamics of human mobility is of great importance in urban management, especially during the COVID-19 pandemic. Traditionally, the human mobility data is collected by roadside sensors, which have limited spatial coverage and are insufficient in large-scale studies. With the maturing of mobile sensing and Internet of Things (IoT) technologies, various crowdsourced data sources are emerging, paving the way for monitoring and characterizing human mobility during the pandemic. This paper presents the authors’ opinions on three types of emerging mobility data sources, including mobile device data, social media data, and connected vehicle data. We first introduce each data source’s main features and summarize their current applications within the context of tracking mobility dynamics during the COVID-19 pandemic. Then, we discuss the challenges associated with using these data sources. Based on the authors’ research experience, we argue that data uncertainty, big data processing problems, data privacy, and theory-guided data analytics are the most common challenges in using these emerging mobility data sources. Last, we share experiences and opinions on potential solutions to address these challenges and possible research directions associated with acquiring, discovering, managing, and analyzing big mobility data.

## Introduction

In December 2019, the coronavirus disease 2019 (COVID-19) was first detected in human beings, which quickly developed into a global pandemic. As of June 11, 2021, the ongoing pandemic has reached 220 countries and territories, causing over 175 million cases and 3.8 million deaths globally, and the number is still increasing (Pettersson et al., 2021). To contain the spread of the disease, different epidemic control measures have been undertaken globally to reduce the transmission rate of COVID-19, such as accelerating the large-scale testing, enhancing clinical management, conducting rapid isolation of confirmed and suspected cases, performing contact tracing, and more importantly, controlling human movement (Bisanzio et al., 2020; Huang et al., 2020; Yechezkel et al., 2021).

Many studies have demonstrated that human mobility is an essential component of respiratory infectious disease transmission, especially in the COVID-19 pandemic. Performing restrictions on human mobility can effectively reduce the transmission rate and protect people from this threat (Kraemer et al., 2020; Pan et al., 2020). Since the pandemic began, various mobility control measures and policies have been implemented at different scales, such as global and national travel bans, regional lockdown and stay-at-home orders, as well as individual-level quarantine, self-isolation, and social distancing. Although these movement-controlling measures worked effectively to mitigate the spread of COVID-19, they also posed significant negative influences on the economy and society (Nouvellet et al., 2021). Studies have shown that massive lockdown measures not only lead to a significant decline in the economy with numerous job losses but also potentially cause pervasive physical and mental health problems for human beings, especially in the vulnerable population groups. The World Health Organization (WHO) also suggests that countries and health authorities should implement targeted movement-controlling interventions based on the local situation when and where needed. While the whole world is looking forward to going back to normal life, it is becoming important to advance the understanding of the relationship between the dynamics of human mobility and the spread of COVID-19. Studies have demonstrated that effectively monitoring the human mobility dynamics during the pandemic could not only benefit modeling the spread and size of epidemics, assessing the effectiveness of ongoing movement-controlling measures, but more importantly, help the government and health authorities to decide whether ease or tighten the mobility restrictions.

Although monitoring human mobility dynamics shows a great importance in fighting against COVID-19, what mobility data sources are available and how well they can reflect the relationship between human mobility and virus transmission still need further investigation. With the advancement of data acquisition and transmission techniques, unprecedented amounts of human mobility data are continually being generated and collected from various data sources, such as social media, roadside sensors, cellular signaling data, GPS-enabled smartphones, and connected vehicles (CVs), among others. These emerging mobility datasets are usually massive in size, spatiotemporally fine-scaled, and high dimensional (e.g., multivariate and multivalued), providing researchers with a rich source of information to monitor the human movements in response to the COVID-19 pandemic. It is worth noting that, although these emerging mobility data sources are promising, they usually show different data characteristics and therefore lead to different applications and limitations, which need to be discussed and documented.

This opinion paper is intended to facilitate the discussion on the utilization of emerging geo-data sources to reveal human mobility dynamics during the COVID-19 pandemic. Although some researchers have provided systemic reviews on the available human mobility data sources in the COVID-19 research (Hu et al., 2021), a more in-depth review and discussion are still needed to further assess the applications of some representative data sources during the pandemic. This paper supplements the existing literature by providing reviews specifically focused on three promising mobility data sources: mobile device data, social media data, and CV data. We selected a representative data source from each type of mobility data (SafeGraph, Twitter, and Wejo) and shared our technical and methodological experiences to utilize them in COVID-19 related research. The opinions on using these data sources are based on the authors’ published and ongoing research works as well as the findings from discussions held in the Symposium on Human Dynamics Research at the American Association of Geographers Annual Meeting 2021. This paper aims to help governments and researchers easily identify available data sources, point out their strengths and limitations in monitoring human movements during the pandemic, as well as share experiences on the applications of emerging geoinformatics technologies to address the technological challenges associated with the acquisition, discovery, management, and analysis of big geospatial data.

## Emerging data sources

To track dynamic human mobility patterns, a prerequisite for measuring human response to the pandemic is the availability of fine-resolution datasets. While entering the “social big data era”, a series of intertwining concepts that include “Web 2.0”, “Citizen as Sensors”, and “Volunteered Geographic Information” lead to the growing popularity of crowdsourced data sources, largely facilitating human mobility monitoring from wider audiences. This section introduces three promising crowdsourced mobility data: mobile device data, social media data, and CV data. We first summarized the most recent COVD-19 related research works based on each type of data. Then, we selected three specific data examples (SafeGraph, Twitter, and Wejo) for each data type to illustrate their generalized data processing flow. Last, we summarized their appropriate applications and limitations in COVID-related studies.

### Mobile device data

As a necessity of people in today’s world, smartphones have served as sensors in capturing data and play key roles in combatting the COVID-19. Companies such as Google, Facebook, Baidu, Apple, Cuebiq, Descartes Labs, and SafeGraph have released their open datasets collected from millions of mobile devices in monitoring human mobility patterns as well as behaviors (e.g., social distancing, shelter-in-place orders). Thanks to their high penetration rates, such records are generally with high representativeness, thus becoming a preferred data source for various COVID-19 studies that demand accurate human spatial interactions (Chang et al., 2021, b; Glaeser et al., 2020). In addition, researchers have also released a series of open-source mobility datasets derived from mobile devices, such as multiscale mobility patterns in the U.S. (Kang et al., 2020), mobility changes in Italy (Pepe et al., 2020), mobility and COVID-19 infections in China (Xu, Gutierrez, et al., 2020). These datasets provide abundant resources that help decipher global human mobility patterns at multiple scales (such as country, state, county, and even census tract/neighborhood) in a frequently updated manner. Several online platforms were built using the aforementioned datasets. One notable effort is by Li et al. (2021, b), who designed an ODT FLOW platform with the capacity to extract, analyze, and share SafeGraph mobility records in response to the soaring needs of human mobility data, especially during disaster events such as the COVID-19 pandemic we are facing. In collaboration with Descartes Labs, Gao et al. (2020) designed a dashboard to present mobility dynamics at the U.S. county-level using mobility records from Descartes Labs.

Taking the SafeGraph data as an example, mobility records from SafeGraph are derived via a panel of GPS points from 45 million anonymous mobile devices (about 10% of mobile devices in the U.S.). SafeGraph provides the mobile phone location data in the CSV format files, convenient for data processing and analysis. By performing clustering algorithms (e.g., density-based spatial clustering of applications with noise [DBSCAN]) on the mobile phone location data, the users’ home locations as well as their visits to various points of interest (POIs) and places can be obtained. Typically, only a cluster of points with a duration of at least 1 min can be retained as a “visit” for each user. By spatially joining the users’ home locations and visiting places to different geographical units (e.g., census tract, county, state), we could obtain the aggregated mobility patterns (origin and destination [OD] pairs) at different spatial scales.

Mobility patterns at different spatial scales derived from mobile devices play a significant role in tracking the dynamics of human mobility and have been used to support government policy decision-making during the pandemic. Researchers have constructed human mobility flow networks for examination in countries such as China, Japan, Italy, France, Chile, and U.K. (Gatto et al., 2020; Jia et al., 2020; Pullano et al., 2020; Yabe et al., 2020), to understand the effect of different lockdown strategies and intervention scenarios in containing the virus spread. In addition, algorithms have been developed to model and simulate disease spread by augmenting human mobility patterns for future infection predictions. For example, Benzell et al. (2020) evaluated the transmission risk of multiple categories of POIs and provided reopening guidelines. Hou et al. (2021) and Thomas et al. (2020) took advantage of fine-scale human mobility datasets and discovered the spatial heterogeneity patterns, largely benefiting the governmental decision-making process. It’s worth noting that the individual-level human trajectory with such details has raised ethical concerns on whether sharing or utilizing them is appropriate, even in a time of crisis. To protect user privacy, most mobile device data are aggregated to the neighborhood level when releasing, so that individual records cannot be traced. The accuracy of the geolocations collected from mobile devices with different GPS quality becomes another concern during the analysis of many mobile datasets. Meanwhile, although SafeGraph’s sampling is highly representative given its high correlation with the actual U.S. census data in various demographic and socioeconomic dimensions (Huang, Lu, et al., 2021), the sampling rates in rural areas and underserved communities are obviously lower than the urban areas. This sampling bias may influence the performance of SafeGraph’s data in tracking underserved communities’ mobility dynamics.

### Social media data

Social media represents the emergence of virtual communities and networks where different users could create, share, and exchange various information. The vast sensing network composed of millions of active social media users serves as a new venue where timely human spatial interactions can be collected, stored, shared, mined, analyzed, and visualized in a rapid manner (Dekel & Shamir, 2009). The valuable user-generated information from social media platforms (often large in volume), when coupled with geo-information, allows human mobility dynamics to be monitored in an active, near-real-time, and less privacy-concerning manner (Huang et al., 2020). Social media has many unique features. Compared to passively collected GPS positions from mobile devices, social media data are less abundant spatiotemporally (owing to their active sharing characteristics) but are less intrusive, more accessible, and more harmonized (Li, Huang, Ye, et al., 2021). The less privacy-concerning nature of social media can be attributed to the user sharing settings. Popular social media platforms include Twitter, WhatsApp, Messenger, Instagram, Facebook, WeChat, Weibo, QQ, Tik Tok, to list a few. However, not all of them open-source their database or permit information mining unless a certain agreement is met.

To retrieve social media records for mobility observation purposes, scholars can either establish connections with companies to obtain their tailored mobility records or use the provided downloading portals, in many cases the Application Programming Interfaces (APIs). However, given the large volume of social media data, special handling approaches are often needed, such as cluster storage, database management, cloud/parallel computing, and multi-thread aggregation. Twitter, for instance, gives privacy control to users, as it allows them to determine whether to share content to the public, whether to reveal locations, and what levels of locational accuracy to be revealed. In addition, Twitter posts provide rich and diverse data sources, including texts, pictures, and videos. Researchers can mine the contextual knowledge and information from Twitter posts through different natural language processing and image segmentation algorithms, benefiting our understanding of stories behinds these trips (e.g., users’ emotional change, social network change).

Mobility records mined from social media platforms have been proved to be one of the important mobility data sources that benefit our understanding of human mobility dynamics during the COVID-19 pandemic. For example, Y. Li et al. (2020) analyzed the mobility pattern during the initial stage of the COVID-19 outbreak in China using the Tencent mobility database derived from various Tencent media platforms. In collaboration with Facebook Data for Good, Chang, Kahn, et al. (2021) adopted Facebook colocation data and Facebook movement data to understand movement patterns and built meta-population models that incorporate human movement data to access the potential effects of local travel restrictions in Taiwan. Zarei et al. (2020) constructed the first Instagram dataset on COVID-19 that involves locational information as one of the features to assist communities in better understanding the mobility and sentimental dynamics. Among all social media platforms that allow mobility data mining, Twitter has become the most popular and the largest source, thanks to its free access to about 1% of its total content (Martín et al., 2020). In early 2021, Twitter released a new academic-oriented Application Programming Interface (API) that grants free access to full-archive search for researchers to obtain more precise, complete, and unbiased data (Twitter, 2021), greatly benefitting future Twitter-based human mobility investigations. Numerous efforts have been made to harvest the geospatial contexts from Twitter posts. One notably effort is by Huang et al. (2020), who harnessed 580 million geotagged tweets worldwide to shed light on the geographically varying difference in policy implementations and discrepancies in policy compliance. Similarly, Bisanzio et al. (2020) took advantage of geospatial contexts from geotagged tweets, aiming to predict the spatiotemporal spread of worldwide reported COVID-19 cases at the initial stage of the COVID-19 outbreak. Xu et al. (2020, b) designed a Twitter social mobility index that measures social distancing compliance and users’ travel behaviors on a weekly basis. Huang, Li, et al. (2021) compared mobility records derived from Twitter with the ones from Google (Google location history service), Apple (Apple map), and Descartes Labs (GPS from mobile devices). Their results reveal a high similarity in mobility dynamics among different data sources at the U.S. county level during the COVID-19 pandemic, suggesting that Twitter data can, to a certain extent, substitute or supplement mobility records collected from other sources. Despite these advantages, several notable issues in social media derived mobility that deserve to be recognized. First, although social media are mode-free (not restricted to certain travel means), the representativeness of social media derived mobility needs further investigation, as studies have shown that they tend to be biassed towards certain age or racial groups and such biases are not geographically-constant (Culotta, 2014; Jiang et al., 2019). Second, social media records are usually with rather sparse spatiotemporal granularity, leading to great difficulty in reconstructing individuals’ detailed trajectories. Thus, certain levels of aggregation are necessary to mitigate the data sparsity (Martín et al., 2021). Third, unlike mobility records derived from GPS pings, the accuracy of geo-information from social media can largely vary and greatly depends on users’ specific settings, posing challenges for comparing and summarizing mobility records with different levels of accuracy.

### Connected vehicle data

CVs are rapidly becoming the new paradigm of road transport, which has been widely believed to influence transportation safety, efficiency, and sustainability positively. CVs represent the unification of various connectivity technologies, enabling the vehicles to communicate with other vehicles (V2V), transportation infrastructures (V2I), and the “Cloud” (V2C) for achieving the goal of “self-driving” (Hoseinzadeh et al., 2020; Talebpour & Mahmassani, 2016). Although most commercially available vehicles are still far from completely automating the driving task, most of them already could monitor the driving environment and vehicle movements through vehicular sensors. Many world-leading auto manufacturers, like Toyota, GM, BMW, Tesla, among others, have ramped up the production of CVs, which could access and transmit vehicular sensors’ data to the cloud (Miles, 2019). Meanwhile, many automotive data companies also emerged to facilitate the utilization of CV data. Like Wejo, Otonomo, Smartcar, Vinili, and CarAlgo, these data companies bridge the data providers—auto manufacturers with data users by ingesting, aggregating, and normalizing the raw CV data and delivering the enriched and organized datasets to end-users (Miles, 2019).

Unlike the aforementioned data sources, the CV data is collected from vehicles, directly reflecting the dynamics of traffic mobility. For example, Wejo, as a leading CV data start-up, provides high-sampling and multi-dimensional vehicle movements and driving event (e.g., hard braking, hard acceleration, speeding) data. This data platform has currently partnered with multiple world-leading auto manufacturers and collected data from millions of vehicles with a sampling rate of 3 s per waypoint. Each waypoint describes the timestamp, location, and movement-related information (e.g., speed, heading) of a vehicle’s trajectory. Wejo claims that their CV data products could access over 90 different vehicular sensors and cover 95% of road networks in the U.S., with about 12 billion data points collected every day at a best temporal resolution of every 3 s. Our preliminary studies in Texas also demonstrated that Wejo data has good spatiotemporal coverage in both urban and rural regions of Texas. CV data shows great superiority in data quality, volume, consistency, and richness compared to traditional mobility data sources, making it a promising data source for monitoring urban mobility dynamics. The CV data is pre-processed by Wejo and delivered to the cloud storage platforms (e.g., Microsoft Azure, AWS Could Storage Services), organized in the Apache Parquet format. The online big data analytic platforms (e.g., Azure Databricks and AWS Databricks) are suitable for processing the big CV dataset. For example, Azure Databricks supports the latest versions of Apache Spark, allowing its users to seamlessly integrate with any open-source libraries and quickly establish a fully managed Apache Spark environment. The clustering computing frameworks for processing large-scale spatial data (e.g., Apache Sedona, GeoMesa) are needed to load, partition, analyze, and visualize the large dataset.

However, to the best of our knowledge, studies on the application and utilization of CV data in mobility monitoring are still underexplored, especially for the mobility changes caused by COVID-19. Wejo’s data science team has utilized their CV data to capture the traffic change across the U.S. since the pandemic began, indicating that the pandemic has led to a 40.7% average decrease of trips in the U.S. since the stay-at-home order came into effect (Wejo, 2021). However, how to comprehensively evaluate the effectiveness of the CV data in response to disease management, as well as how to systemically utilize the CV data in different movement-controlling measures assessment and disease transmission modeling, still need to be further explored. It is worth noting that the CV shows exponentially greater requirements in data storage and computation due to its massive data size. For example, our data evaluation shows the one-month CV movement data in Texas contains 108.19 million trips collected from more than 1 million vehicles with a size of around three terabytes. Therefore, advanced data storage and computing techniques (e.g., cloud computing, distributed computing, and serverless computing) are needed for effectively managing and manipulating this emerging mobility big data. Meanwhile, although directly collecting data from vehicles can ensure data purity and quality, it also limits the application of this data only to vehicle movements, thus the mobility of active transportation (e.g., biking, walking) cannot be monitored through this data.

## From emerging mobility data to GeoInformatics: challenges and potential solutions

The emergence of these crowdsourced mobility data sources marks the evolution of the geospatial research paradigm into a new era of geoinformatics. With the recent proliferation of the Internet of Things (IoT), Internet of Everything (IoE), and Information and Communications Technology (ICT), various big data sources are becoming available, which enable situational awareness and informed simulations to generate holistic understanding, hypothesis testing, and data-driven insights into the various social demands, behaviors, and dynamics in urban and rural areas. The rapid increase in the volume, variety, and velocity of multi-domain datasets often allows urban planners and scientists to analyze particular issues at multiple scales. Meanwhile, the tremendous amount of geo-data collected from a wide spectrum of sources are often heterogeneous and unstructured, which entails data quality issues, presenting different types of challenges to geospatial research efforts. Thakuriah et al. (2017) described these challenges as the “big data tsunami” (Laney, 2001) and categorized them into four types, namely (1) technological, (2) methodological, (3) social & political, and (4) theoretical and epistemological. Technological challenges are often associated with the limitations of the technology (e.g., storage, computational speed, and internet bandwidth), which do not entail gaps and unknowns in the domain knowledge. On the other hand, methodological challenges are caused by the gap of domain knowledge and expertise, such as the data uncertainty resulting from the design of the method and experiment. Social and political challenges are often originated from political, legal, and ethical concerns, such as data privacy and locality. Laws and regulations in many states, countries, or governmental agencies, such as the United Kingdom Data Protection Act (Jaar & Zeller, 2009), the Swiss Federal Act on Data Protection (Staiger, 2020), and the Canadian Personal Information Protection and Electronic Documents Act (Harbour et al., 2003), regulate that sensitive or confidential information should not leave the physical boundaries of the country or region (residency), or should not be exposed to unauthorized parties (privacy). Many of these regulations put restrictions on the acquisition and transfer of social sensing and public datasets for fair use purposes and scientific applications. The theoretical and epistemological challenges are linked with whether the researcher could build an appropriate interrelationship between their epistemological and theoretical stances to understand the question and the methodology they adopted.

In the context of exploring urban mobility patterns under the influences of the COVID-19 pandemic, we summarized the major data challenges from the technological, methodological, and social & political perspectives based on the previous review and knowledge in the geoinformatics disciplines for each data type (Table 1). The theoretical and epistemological challenges in analyzing the aforementioned types of mobility data are common and associated with the inappropriate interpretation of the data and analytical results. Most existing studies derive causal inference, insights, and generate hypotheses solely based on data but don’t have a solid fusion between the human mobility domain knowledge and data analytics. Therefore, we didn’t list it in this table.

**Table 1:** Challenges by type of data

Data Type	Challenge Types	Challenges
Mobile Device	Technical	Data coverage in spatial and temporal dimensions may vary dramatically and is limited by the quality of the mobile network.
	Methodological	The accuracy and reliability of the geolocation are a concern due to the appropriateness of data-sharing devices and processes. The OD flows for each user are estimated by clustering the users’ staying points. Different clustering methods and selection criteria could lead to different results. The OD flows obtained from mobile devices need to be spatially aggregated to different geographic units to reveal the dynamics of human mobility at different spatial scales.
	Social and political	Data privacy concerns prevent the sharing of individual user records. Only spatially aggregated information is available.
Social Media	Technical	Accuracy of geo-information varies across different social media platforms, user settings, and mobile devices.
	Methodological	Data bias from different user groups (e.g., races, regions, and age.) Social media data is more heterogeneous with high variability of data types, formats, and qualities.
	Social and political	The locational information in social media data should not be used to identify individual users.
Connected Vehicle (CV)	Technical	The tremendous volume and rapid data collection speeds of CV data lead to rigorous requirements for computing and storage devices. The spatiotemporal coverage and availability of the CV data vary across regions.
	Methodological	Different CV data companies are partnered with different original equipment manufacturers (OEMs) to collect CV data from a variety of vehicles. The data collection and processing also vary from OEM to OEM, leading to severe data uncertainty. Meanwhile, only vehicle movements are covered by CV data; the mobility of active transportation (e.g., biking, walking) is missing.
	Social and political	The high detailed trajectory and driving behavior datasets collected from CVs may have the risk of revealing too much personal information (e.g., home and working addresses.)

As summarized in Table 1, most of the technical and methodological challenges are associated with two keywords: “big data processing” and “data uncertainty”. Tackling these challenges often requires researchers to develop proficient skills and knowledge in computer sciences and data sciences and spend a significant amount of time developing software and web tools for processing and archiving various types of mobility data. With the recent evolution in data science products, many generalizable cyberinfrastructures and big-data platforms developed by both the commercial and open-source communities can be adapted to resolve big data processing challenges in mobility research.

At the technical level, many of these products are cloud-scale applications developed using the state-of-art computing and storage paradigm (e.g., mobile edge computing, fog computing, and distributed data stores), providing intuitive web-style interfaces and visual dashboards to allow users to search, discover, explore, and perform analytics (through machine learning and visualizations) on various IoT-connected data sources in near-real-time with minimum programming efforts. An example of these products would be the Elastic Stack, a combination of open-source web-based data science products (Elasticsearch, Logstash, and Kibana) from Elastic (Fakhir, 2018), which is designed to allow users without intensive big-data and coding expertise, through an end-to-end workflow, to discover data from any type and format through Elasticsearch engine, processing collected data using Logstash pipeline, and analyze and visualize that data in real-time through the Kibana online platform, which is powered by a variety of modular data analytics and visualization libraries. Recently, the Elastic Stack has been increasingly applied to build data-driven research applications that analyze both COVID-19 and mobility data (Cecchet et al., 2020; Thakur et al., 2020), severing as an effective tool to lower the technical barriers for addressing big data challenges. Data products that offer similar capabilities as the Elastic Stack include Datadog, Grafana, and Splunk. As for the data uncertainty associated with the crowdsourced mobility data, it can be managed and analyzed by imposing system-based metadata standards that could help data scientists identify records retrieved using devices without reliable GPS or under poor network quality.

At the methodological level, a comprehensive ontology-driven approach could be devised to further improve the description of different mobility datasets (e.g., types, characteristics, challenges and limitations, and computational resources required for data storage and analysis). The ontology should comply with well-known international standards for data description, to enhance the visibility and searchability of novel data sources across the internet, as well as to automate the integration and handling of heterogeneous datasets of different resolutions and types. Time-space geography theorems, such as the space-time prism model, could be potentially applied to derive individual movement trajectories from mobile device data that is aggregated to the neighborhood level through the determination of likelihood of human presence based on time constraints, as well as GIS data which defines impassable areas (e.g., roof, bushes, and water space).

The theoretical and epistemological challenges could be addressed through the theory-guided data analytics approach, which aims to bring domain experts and their knowledge and experiences into the data-driven analytics to enable rational interpretation of insights, patterns, and inferences derived from various mobility data. As for social and political challenges, some solutions are already proposed to protect individuals’ data confidentiality and privacy such as anonymization, data obfuscation, cryptographic mechanisms, compensating users for privacy loss, among others (Halder, 2017). But, more importantly, we believe standardized guidelines and regulations for crowdsourced data collection and utilization need to be proposed by the joint efforts from both the public and private sectors, research communities, as well as government authorities.

## Concluding remarks and vision—promoting theory-driven research and keeping humans in the loop

The emerging geospatial mobility data plays a vital role in the exploration of human mobility patterns and dynamics in response to the COVID-19 pandemics. To ensure the extraction of useful insights and inferences, different types of emerging mobility datasets, which include data collected from mobile devices, social media, and CV, should be appropriately analyzed and handled with the consideration of their research benefits and limitations. This opinion paper provided in-depth reviews of current advances in COVID-19 mobility research developed based on each type of emerging data and discussed their potential research opportunities and limitations. We summarized the technical blockers and challenges associated with the effective analysis of these emerging data types, followed by the sharing of our experiences on addressing these challenges through emerging urban informatics products and techniques.

The recent smart mobility and smart city initiatives have introduced many novelties in social sensing and connected sensor systems, as well as data-driven techniques for exploring human mobility phenomena that are conceptually complex and computationally intensive to analyze and model using theory-driven approaches. We note that the theory-guided data analytics approaches would be a major trend in future mobility research, through which big-data analytics can be validated and interpreted using domain theory and knowledge, while theory-driven techniques (e.g., process-based models) can be retrofitted to incorporate a new variety of mobility data. Previous geoinformatics reviews conclude that the need for a wide variety of computer and data science skills would be critical for conducting future mobility research, while we noted that many web-based data-analytics platforms and cyberinfrastructure developed by the industry sector could be used to lower the technical barriers and requirements to data-centric mobility research. Many data-analytics platforms also provide intuitive user workflows coupled with abstracted data visualizations to allow non-expert users to explore how data model parameters could affect the results and the performances of different data models (machine learnings and statistics) in performing the same data analysis. Our opinion would be that many data science products developed by industry could be readily applied to tackle big-data challenges in mobility research, saving urban scientists time and efforts on creating data analytics tools from scratch and ensuring better data and software interoperability between different research efforts. More recent approach trends to incorporate the human components (e.g., public awareness, engagement, and analytical reasoning) into the technology-driven pipeline automated for the data acquisition, discovery, processing, and analysis to facilitate the heuristic exploration of human mobility dynamics in complex and multidimensional metropolitan areas. Under this general trend, the scientific gamification approach has been increasingly reported in mobility research to involve humans (e.g., the general public, policymakers) in the data analysis, planning, and decision support processes in the form of serious games to effectively collect user data (e.g., social demands, opinion, and needs).

## Data Availability

Not applicable.

## References

[CR1] Benzell, S. G., Collis, A., & Nicolaides, C. (2020). Rationing social contact during the COVID-19 pandemic: Transmission risk and social benefits of US locations. *Proceedings of the National Academy of Sciences of the United States of America, 117*(26), 14642–14644. 10.1073/pnas.2008025117 32522870 10.1073/pnas.2008025117 PMC7334565

[CR2] Bisanzio, D., Kraemer, M. U. G., Bogoch, I. I., Brewer, T., Brownstein, J. S., & Reithinger, R. (2020). Use of twitter social media activity as a proxy for human mobility to predict the spatiotemporal spread of COVID-19 at global scale. *Geospatial Health, 15*(1). 10.4081/gh.2020.882 10.4081/gh.2020.882 32575957

[CR3] Cecchet, E., Acharya, A., Molom-Ochir, T., Trivedi, A., & Shenoy, P. (2020). WiFiMon: A mobility analytics platform for building occupancy monitoring and contact tracing using wifi sensing: Poster abstract. In *SenSys 2020 - proceedings of the 2020 18th ACM conference on embedded networked sensor systems*. 10.1145/3384419.3430598

[CR4] Chang, M. C., Kahn, R., Li, Y. A., Lee, C. S., Buckee, C. O., & Chang, H. H. (2021). Variation in human mobility and its impact on the risk of future COVID-19 outbreaks in Taiwan. *BMC Public Health, 21*(1), 226. 10.1186/s12889-021-10260-7 33504339 10.1186/s12889-021-10260-7 PMC7838857

[CR5] Chang, S., Pierson, E., Koh, P. W., Gerardin, J., Redbird, B., Grusky, D., & Leskovec, J. (2021). Mobility network models of COVID-19 explain inequities and inform reopening. *Nature, 589*(7840), 82–87. 10.1038/s41586-020-2923-3 33171481 10.1038/s41586-020-2923-3

[CR6] Culotta, A. (2014). Reducing sampling Bias in social media data for county health inference. In *Joint Statistical Meetings Proceedings*.

[CR7] Dekel, O., & Shamir, O. (2009). Vox populi: Collecting high-quality labels from a crowd. In *COLT 2009 - the 22nd conference on learning theory*.

[CR8] Fakhir, R. (2018). Architecture Analysis and Design based on Elasticsearch and Kibana to Process and Visualize near Real-Time Data [the University of Applied Sciences FH Campus Wien]. https://pub.fh-campuswien.ac.at/obvfcwhsacc/content/titleinfo/2800584/full.pdf

[CR9] Gao, S., Rao, J., Kang, Y., Liang, Y., & Kruse, J. (2020). Mapping county-level mobility pattern changes in the United States in response to COVID-19. *SIGSPATIAL Special, 12*(1), 16–26. 10.1145/3404820.3404824

[CR10] Gatto, M., Bertuzzo, E., Mari, L., Miccoli, S., Carraro, L., Casagrandi, R., & Rinaldo, A. (2020). Spread and dynamics of the COVID-19 epidemic in Italy: Effects of emergency containment measures. *Proceedings of the National Academy of Sciences of the United States of America, 117*(19), 10484–10491. 10.1073/pnas.2004978117 32327608 10.1073/pnas.2004978117 PMC7229754

[CR11] Glaeser, E. L., Gorback, C., & Redding, S. J. (2020). How much does COVID-19 increase with mobility? In *Evidence from New York and four other US cities (No. 27519)* http://www.nber.org/papers/w27519 10.1016/j.jue.2020.103292 PMC7577256 33106711

[CR12] Halder, B. (2017). Privacy, Security and Data Protection in Crowdsourcing Platforms: Issues and Recommendations. *SSRN Electronic Journal*. 10.2139/ssrn.3022566

[CR13] Harbour, L. J., MacDonald, I. D., & Gill, E. (2003). Protection of personal data: The United Kingdom perspective the privacy project. *Defense Counsel Journal, 70*(1), 99–105 https://heinonline.org/HOL/P?h=hein.journals/defcon70&i=101

[CR14] Hoseinzadeh, N., Arvin, R., Khattak, A. J., & Han, L. D. (2020). Integrating safety and mobility for pathfinding using big data generated by connected vehicles. *Journal of Intelligent Transportation Systems: Technology, Planning, and Operations*. 10.1080/15472450.2019.1699077

[CR15] Hou, X., Gao, S., Li, Q., Kang, Y., Chen, N., Chen, K., Rao, J., Ellenberg, J. S., & Patz, J. A. (2021). Intracounty modeling of COVID-19 infection with human mobility: Assessing spatial heterogeneity with business traffic, age, and race. *Proceedings of the National Academy of Sciences, 118*(24), e2020524118. 10.1073/pnas.2020524118 10.1073/pnas.2020524118 PMC8214685 34049993

[CR16] Hu, T., Wang, S., She, B., Zhang, M., Huang, X., Cui, Y., Khuri, J., Hu, Y., Fu, X., Wang, X., Wang, P., Zhu, X., Bao, S., Guan, W., & Li, Z. (2021). Human mobility data in the COVID-19 pandemic: Characteristics, applications, and challenges. *International Journal of Digital Earth, 14*(9), 1126–1147. 10.1080/17538947.2021.1952324

[CR17] Huang, X., Li, Z., Jiang, Y., Li, X., & Porter, D. (2020). Twitter reveals human mobility dynamics during the COVID-19 pandemic. *PLoS ONE, 15*(11 November), e0241957. 10.1371/journal.pone.0241957 33170889 10.1371/journal.pone.0241957 PMC7654838

[CR18] Huang, X., Li, Z., Jiang, Y., Ye, X., Deng, C., Zhang, J., & Li, X. (2021). The characteristics of multi-source mobility datasets and how they reveal the luxury nature of social distancing in the U.S. during the COVID-19 pandemic. *International Journal of Digital Earth, 14*(4), 424–442. 10.1080/17538947.2021.1886358

[CR19] Huang, X., Lu, J., Gao, S., Wang, S., Liu, Z., & Wei, H. (2021). Staying at home is a privilege: Evidence from fine-grained mobile phone location data in the United States during the COVID-19 pandemic. *Annals of the American Association of Geographers, 0*(0), 1–20. 10.1080/24694452.2021.1904819

[CR20] Jaar, D., & Zeller, P. E. (2009). Canadian privacy law: The personal information protection and electronic documents act (PIPEDA). *International In-House Counsel Journal, 2*(7), 1135–1146 https://heinonline.org/HOL/P?h=hein.journals/iihcj2&i=487

[CR21] Jia, J. S., Lu, X., Yuan, Y., Xu, G., Jia, J., & Christakis, N. A. (2020). Population flow drives spatio-temporal distribution of COVID-19 in China. *Nature, 582*(7812), 389–394. 10.1038/s41586-020-2284-y 32349120 10.1038/s41586-020-2284-y

[CR22] Jiang, Y., Li, Z., & Ye, X. (2019). Understanding demographic and socioeconomic biases of geotagged twitter users at the county level. *Cartography and Geographic Information Science, 46*(3), 228–242. 10.1080/15230406.2018.1434834

[CR23] Kang, Y., Gao, S., Liang, Y., Li, M., Rao, J., & Kruse, J. (2020). Multiscale dynamic human mobility flow dataset in the U.S. during the COVID-19 epidemic. *Scientific Data, 7*(1), 1–13. 10.1038/s41597-020-00734-5 33184280 10.1038/s41597-020-00734-5 PMC7661515

[CR24] Kraemer, M. U. G., Yang, C.-H., Gutierrez, B., Wu, C.-H., Klein, B., Pigott, D. M., du Plessis, L., Faria, N. R., Li, R., Hanage, W. P., Brownstein, J. S., Layan, M., Vespignani, A., Tian, H., Dye, C., Pybus, O. G., & Scarpino, S. V. (2020). The effect of human mobility and control measures on the COVID-19 epidemic in China. *Science, 368*(6490), 493 LP–493497. 10.1126/science.abb4218 32213647 10.1126/science.abb4218 PMC7146642

[CR25] Laney, D. (2001). 3D data management: controlling data volume, velocity and variety META. *Grp. Resear. Note, 6*. https://scholar.google.com/scholar_lookup?title=3D%20Data%20Management%3A%20Controlling%20Data%20Volume%2C%20Velocity%2C%20and%20Variety&publication_year=2001&author=D.%20Laney.

[CR26] Li, Y., Zeng, Y., Liu, G., Lu, D., Yang, H., Ying, Z., Hu, Y., Qiu, J., Zhang, C., Fall, K., Fang, F., Valdimarsdóttir, U. A., Zhang, W., & Song, H. (2020). Public awareness, emotional reactions and human mobility in response to the COVID-19 outbreak in China- a population-based ecological study. *Psychological Medicine*, 1–8. 10.1017/S003329172000375X 10.1017/S003329172000375X PMC7542325 32972473

[CR27] Li, Z., Huang, X., Hu, T., Ning, H., Ye, X., Huang, B., Li, X., Yang, C. (2021) ODT FLOW: Extracting analyzing and sharing multi-source multi-scale human mobility. *PLOS ONE, 16*(8), e0255259. 10.1371/journal.pone.0255259. 10.1371/journal.pone.0255259 PMC8341631 34351973

[CR28] Li, Z., Huang, X., Ye, X., Jiang, Y., Martin, Y., Ning, H., Hodgson, M. E., & Li, X. (2021). Measuring global multi-scale place connectivity using geotagged social media data. *Scientific Reports, 11*(1), 14694. 10.1038/s41598-021-94300-7 34282241 10.1038/s41598-021-94300-7 PMC8290042

[CR29] Martín, Y., Cutter, S., & Zhenlong, L. (2020). Bridging twitter and survey data for evacuation assessment of Hurricane Matthew and Hurricane Irma. *Natural Hazards Review, 21*(2), 4020003. 10.1061/(ASCE)NH.1527-6996.0000354

[CR30] Martín, Y., Li, Z., Ge, Y., & Huang, X. (2021). *Introducing twitter daily estimates of residents and non-residents at the county level*. 10.3390/socsci10060227

[CR31] Miles, S. (2019). 6 Automotive Data Services Platforms. https://streetfightmag.com/2019/08/23/6-automotive-data-services-platforms/#.YMlWW_lKguU

[CR32] Nouvellet, P., Bhatia, S., Cori, A., Ainslie, K. E. C., Baguelin, M., Bhatt, S., Boonyasiri, A., Brazeau, N. F., Cattarino, L., Cooper, L. V., Coupland, H., Cucunuba, Z. M., Cuomo-Dannenburg, G., Dighe, A., Djaafara, B. A., Dorigatti, I., Eales, O. D., van Elsland, S. L., Nascimento, F. F., et al. (2021). Reduction in mobility and COVID-19 transmission. *Nature Communications, 12*(1), 1–9. 10.1038/s41467-021-21358-2 10.1038/s41467-021-21358-2 PMC7889876 33597546

[CR33] Pan, Y., Darzi, A., Kabiri, A., Zhao, G., Luo, W., Xiong, C., & Zhang, L. (2020). *Quantifying human mobility behaviour changes during the COVID-19 outbreak in the United States*. 10.1038/s41598-020-77751-2 10.1038/s41598-020-77751-2 PMC7691347 33244071

[CR34] Pepe, E., Bajardi, P., Gauvin, L., Privitera, F., Lake, B., Cattuto, C., & Tizzoni, M. (2020). COVID-19 outbreak response, a dataset to assess mobility changes in Italy following national lockdown. *Scientific Data, 7*(1), 230. 10.1038/s41597-020-00575-2 32641758 10.1038/s41597-020-00575-2 PMC7343837

[CR35] Pettersson, H., Manley, B., & Hernandez, S. (2021). *Tracking Covid-19’s global spread*. CNN Health https://edition.cnn.com/interactive/2020/health/coronavirus-maps-and-cases/

[CR36] Pullano, G., Valdano, E., Scarpa, N., Rubrichi, S., & Colizza, V. (2020). Population mobility reductions during COVID-19 epidemic in France under lockdown. *MedRxiv.*10.1101/2020.05.29.20097097 10.1016/S2589-7500(20)30243-0 PMC7598368 33163951

[CR37] Staiger, D. N. (2020). *Swiss data protection law*. 10.1007/978-3-030-28049-9_16

[CR38] Talebpour, A., & Mahmassani, H. S. (2016). Influence of connected and autonomous vehicles on traffic flow stability and throughput. *Transportation Research Part C: Emerging Technologies, 71*. 10.1016/j.trc.2016.07.007

[CR39] Thakur, G., Sparks, K., Berres, A., Tansakul, V., Chinthavali, S., Whitehead, M., Schmidt, E., Xu, H., Fan, J., Spears, D., & Cranfill, E. (2020). COVID-19 joint pandemic modeling and analysis platform. In *Proceedings of the 1st ACM SIGSPATIAL international workshop on modeling and understanding the spread of COVID-19, COVID-19 2020*. 10.1145/3423459.3430760

[CR40] Thakuriah, P. V., Tilahun, N. Y., & Zellner, M. (2017). *Big data and urban informatics: Innovations and challenges to urban planning and knowledge discovery*. Springer Geography. 10.1007/978-3-319-40902-3_2

[CR41] Thomas, L. J., Huang, P., Yin, F., Luo, X. I., Almquist, Z. W., Hipp, J. R., & Butts, C. T. (2020). Spatial heterogeneity can lead to substantial local variations in COVID-19 timing and severity. *Proceedings of the National Academy of Sciences of the United States of America, 117*(39), 24180–24187. 10.1073/pnas.2011656117 32913057 10.1073/pnas.2011656117 PMC7533653

[CR42] Twitter. (2021). *Twitter products for academic researchers | twitter developer*. Twitter https://developer.twitter.com/en/solutions/academic-research/products-for-researchers

[CR43] Wejo. (2021). Wejo COVID-19 Insights. https://www.wejo.com/journey-intelligence/hub

[CR44] Xu, B., Gutierrez, B., Mekaru, S., Sewalk, K., Goodwin, L., Loskill, A., Cohn, E. L., Hswen, Y., Hill, S. C., Cobo, M. M., Zarebski, A. E., Li, S., Wu, C. H., Hulland, E., Morgan, J. D., Wang, L., O’Brien, K., Scarpino, S. V. V., Brownstein, J. S., et al. (2020). Epidemiological data from the COVID-19 outbreak, real-time case information. *Scientific Data, 7*(1), 106. 10.1038/s41597-020-0448-0 32210236 10.1038/s41597-020-0448-0 PMC7093412

[CR45] Xu, P., Dredze, M., & Broniatowski, D. A. (2020). The twitter social mobility index: Measuring social distancing practices with geolocated tweets. *Journal of Medical Internet Research, 22*(12), e21499. 10.2196/21499 33048823 10.2196/21499 PMC7717895

[CR46] Yabe, T., Tsubouchi, K., Fujiwara, N., Wada, T., Sekimoto, Y., & Ukkusuri, S. V. (2020). Non-compulsory measures sufficiently reduced human mobility in Tokyo during the COVID-19 epidemic. *Scientific Reports, 10*(1), 18053. 10.1038/s41598-020-75033-5 33093497 10.1038/s41598-020-75033-5 PMC7581808

[CR47] Yechezkel, M., Weiss, A., Rejwan, I., Shahmoon, E., Ben-Gal, S., & Yamin, D. (2021). Human mobility and poverty as key drivers of COVID-19 transmission and control. *BMC Public Health, 21*(1), 1–13. 10.1186/s12889-021-10561-x 33765977 10.1186/s12889-021-10561-x PMC7993906

[CR48] Zarei, K., Farahbakhsh, R., Crespi, N., & Tyson, G. (2020). *A first instagram dataset on COVID-19* (pp. 2–5) http://arxiv.org/abs/2004.12226

